# 22370 Mechanical Analysis of Posterior Spinal Fusion Assemblies Intended to Cross the Cervicothoracic Junction

**DOI:** 10.1017/cts.2021.571

**Published:** 2021-03-30

**Authors:** John T. Sherrill, David B. Bumpass, Erin M. Mannen

**Affiliations:** 1 University of Arkansas for Medical Sciences; 2 Boise State University

## Abstract

ABSTRACT IMPACT: A comparative evaluation of the mechanical properties of commonly used posterior spinal fusion assemblies will allow surgeons to choose an assembly based on desired properties. The results will better inform surgical decision making and may lead to improved patient outcomes. OBJECTIVES/GOALS: The objective of this study is to evaluate and compare the mechanical properties of three posterior spinal fusion assemblies commonly used to cross the cervicothoracic junction. Fusion success depends on immobilization of vertebrae. The results will better inform surgical decision making and may improve patient outcomes. METHODS/STUDY POPULATION: Three titanium alloy posterior spinal fusion assemblies intended to cross the cervicothoracic junction underwent static compressive bending, tensile bending, and torsion as described in ASTM F1717 to a torque of 2.5 Nm: 3.5mm rods (Assembly A), 3.5mm to 5.5mm dilating rods (Assembly B), and two 3.5mm rods connected to two 5.5mm rods (Assembly C). Five samples of each assembly were attached to ultrahigh molecular weight polyethylene blocks via multiaxial screws for testing. The distance from the axis of rotation to the point of attachment of the rod and cervical screw was used as the lever arm to calculate the force required to create the desired torque for each test: lever arm of 37mm, requiring 67.6N of force to generate 2.5Nm of torque. Force and displacement were recorded, and stiffness of each construct calculated. RESULTS/ANTICIPATED RESULTS: An ANOVA was performed and resulted in p-values all p<0.005, indicating the test groups were significantly different. Therefore, pairwise t-tests with Bonferroni corrections (p<0.017) were used to determine pairs that were significantly different. Assembly A (3.5mm rods only) was found to be significantly less stiff than Assembly B (dilating rods) and Assembly C (3.5mm-connector-5.5mm rods) in each mode of bending: compression bending, tension bending, and torsion. Assembly A had a significantly greater range of motion in compression bending and torsion, but not tension bending, when compared to Assembly B and Assembly C. The only significant difference between Assembly B and Assembly C was found in the stiffness value of compression bending. DISCUSSION/SIGNIFICANCE OF FINDINGS: The results of this study indicate that incorporating a 5.5mm rod in a fusion assembly adds significant stiffness to the posterior spinal fusion construct. When stability of a fusion is of heightened concern, as demonstrated by the ASTM F1717 vertebectomy (worst case scenario) model, including 5.5mm rods increases the likelihood of fusion success.

